# Crystal structure, computational study, and Hirshfeld analysis of *exo*-1,2,3,5-tetra­phenyl-1a’,9b’-di­hydro­spiro­[bi­cyclo­[3.1.0]hexane-6,1′-cyclo­propa[*l*]phenanthren]-2-en-4-one

**DOI:** 10.1107/S2056989025004414

**Published:** 2025-05-30

**Authors:** Alexander D. Roth, Dasan M. Thamattoor

**Affiliations:** a Department of Chemistry Colby College,Waterville ME 04901 USA; University of Massachusetts Dartmouth, USA

**Keywords:** crystal structure, Spiro­penta­ne, Stereochemistry, Hirshfeld analysis

## Abstract

The reaction of dibenzonorcarynyliden(e/oid) with phencyclone was recently reported to give a congested spiro­pentane with *endo* stereochemistry. Herein we report that, in sharp contrast, an analogous reaction using tetra­cyclone, instead of phencyclone, gives the highly crowded title spiro­pentane but with *exo* stereochemistry as determined by X-ray crystallography. This new tetra­cyclone adduct (C_44_H_30_O) crystallizes upon slow evaporation from hexa­nes/ethyl acetate in the monoclinic crystal system and *P*2_1_/*n* (No. 14) space group. It has one mol­ecule in the asymmetric unit and four mol­ecules per unit cell. DLPNO-CCSD(T)/def2-TZVP//B3LYP/def2-SVP calculations indicate that the *endo* spiro­pentane diastereomers from phencyclone and tetra­cyclone are both more stable than the corresponding *exo* forms by 6.68 and 5.35 kcal mol^−1^, respectively. As noted previously in the phencyclone system, favorable π-stacking inter­actions between the two flat biphenyl moieties in the product and transition state may lead to the preferential formation of the *endo* diastereomer. However, the ability of the phenyl rings in the 3,4-position of the tetra­cyclone component to rotate could introduce destabilizing steric inter­actions in the transition state that hinder formation of the *endo* diastereomer in favor of the less thermodynamically stable *exo* isomer.

## Abstract 2

The reaction of dibenzonorcarynyliden(e/oid) with phencyclone was recently reported to give a congested spiro­pentane with *endo* stereochemistry. Herein it is reported that, in sharp contrast, an analogous reaction using tetra­cyclone, instead of phencyclone, gives the highly crowded title spiro­pentane but with *exo* stereochemistry as determined by X-ray crystallography.

## 1. Chemical context

Recently, we disclosed that the treatment of 1,1-di­bromo-1a,9b-di­hydro-1*H*-cyclo­propa[*l*]phenanthrene (**1**) with butyl­lithium at low temperatures followed by quenching with phencyclone (**2**) gave the congested spiro­pentane **3** as the *endo* diastereomer (Roth & Thamattoor, 2024 ▸). Compound **3** presumably issues from trapping the carben(e/oid) derived from **1** with **2**. Conspicuously, the *exo* diastereomer of **3**, the spiro­pentane **4**, was not observed in the reaction. Herein, we report the curious finding that when the trapping agent **2** is replaced by tetra­cyclone (**5**), a decidedly different outcome is observed. In this case, it is the *exo* diastereomer of 1,2,3,5-tetra­phenyl-1a′,9b′-di­hydro­spiro­[bi­cyclo­[3.1.0]hexane-6,1′-cyclo­propa[*l*]phenanthren]-2-en-4-one (**6**) that is found in the reaction mixture. (An alcohol, which is likely produced by addition of the initially formed li­thio­anion to **5** followed by work up, is also formed as a byproduct.) Inter­estingly, we did not observe **7**, the *endo* diastereomer of **6**, in the reaction mixture. The scheme below shows the synthesis of *endo*- and *exo*-spiro­penta­nes **3** and **6**, respectively.
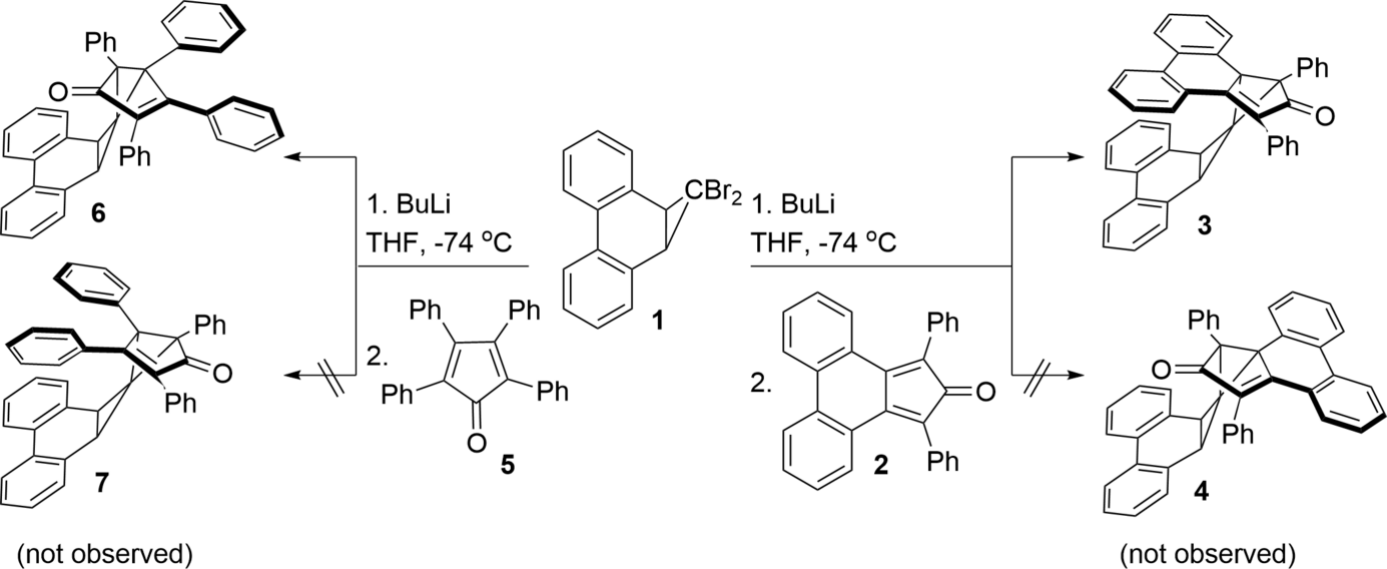


Calculations at the DLPNO-CCSD(T)/def2-TZVP//B3LYP/def2-SVP level of theory (Neese *et al.*, 2020 ▸; Weigend & Ahlrichs, 2005 ▸; Weigend, 2006 ▸; Becke, 1988 ▸; Becke, 1993 ▸; Riplinger & Neese, 2013 ▸; Riplinger *et al.*, 2016 ▸; Riplinger *et al.*, 2013 ▸) indicated that the *endo* spiro­pentane adduct **7** is 5.35 kcal mol^−1^ more stable than its *exo* isomer **6**. To compare, our previous calculations indicated that **3** is more stable than **4** by 6.68 kcal mol^−1^. Thus, the endo diastereomer is calculated to be the more thermodynamically stable product in both cases, although the difference is slightly less for the **6**/**7** pair. We reasoned that the favorable π-stacking inter­actions between the two flat biphenyl moieties in the transition state leading up to the *endo* diastereomer, was likely why **3** was preferred over **4**. In other words, **3** was both the thermodynamic and kinetic product. In the reaction using **5** as the trapping agent, however, the ability of the phenyl rings in the 3,4-position of the dienone component to rotate could introduce destabilizing steric inter­actions that hinder formation of *endo* diastereomer **7** and favor the less thermodynamically stable *exo* isomer **6**.

## 2. Structural commentary

**Figure 1:**
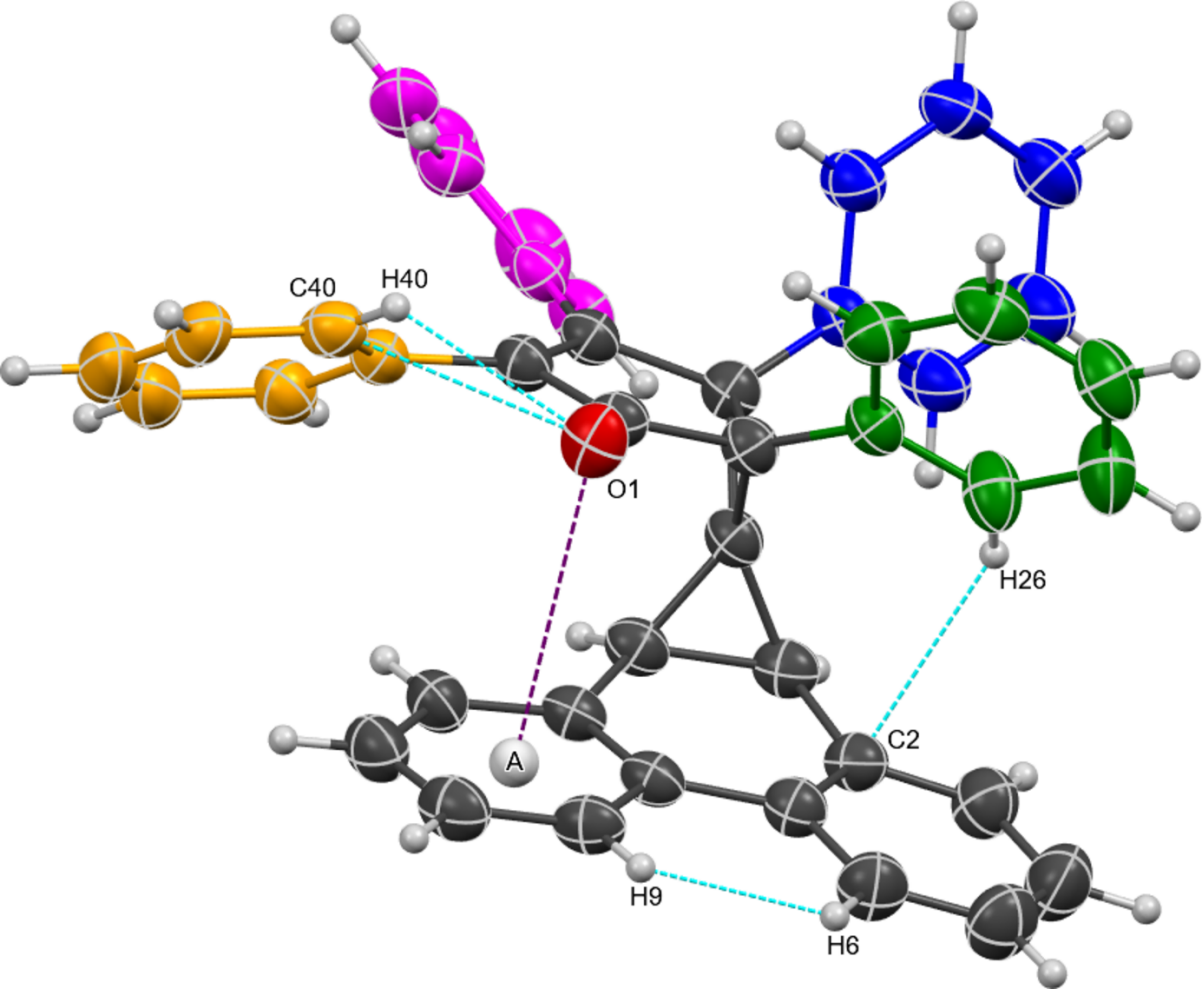
Single-crystal X-ray structure of **6**. Displacement ellipsoids are shown at the 50% probability level.

The crystal structure of **6** is shown in Fig. 1 ▸. The crystal system is monoclinic and belongs to the *P*2_1_/*n* (14) space group with one mol­ecule in the asymmetric unit. The carbonyl group is perched over the erstwhile phenanthrene framework with the oxygen at a distance of 3.472 (2) Å to the centroid marked *A* in Fig. 1 ▸ (purple line). Four intra­molecular short contacts between atoms (sum of vdW radii − 0.3 Å) were also identified (Table 1 ▸) and are designated by the cyan lines in Fig. 1 ▸. The four phenyl rings attached to the cyclo­pentenone moiety are all non-coplanar with the five-membered ring as listed in Table 2 ▸. The blue ring shows the largest twist [73.67 (10)°] and the magenta ring has the smallest [35.06 (9)°].

## 3. Supra­molecular features

**Figure 2:**
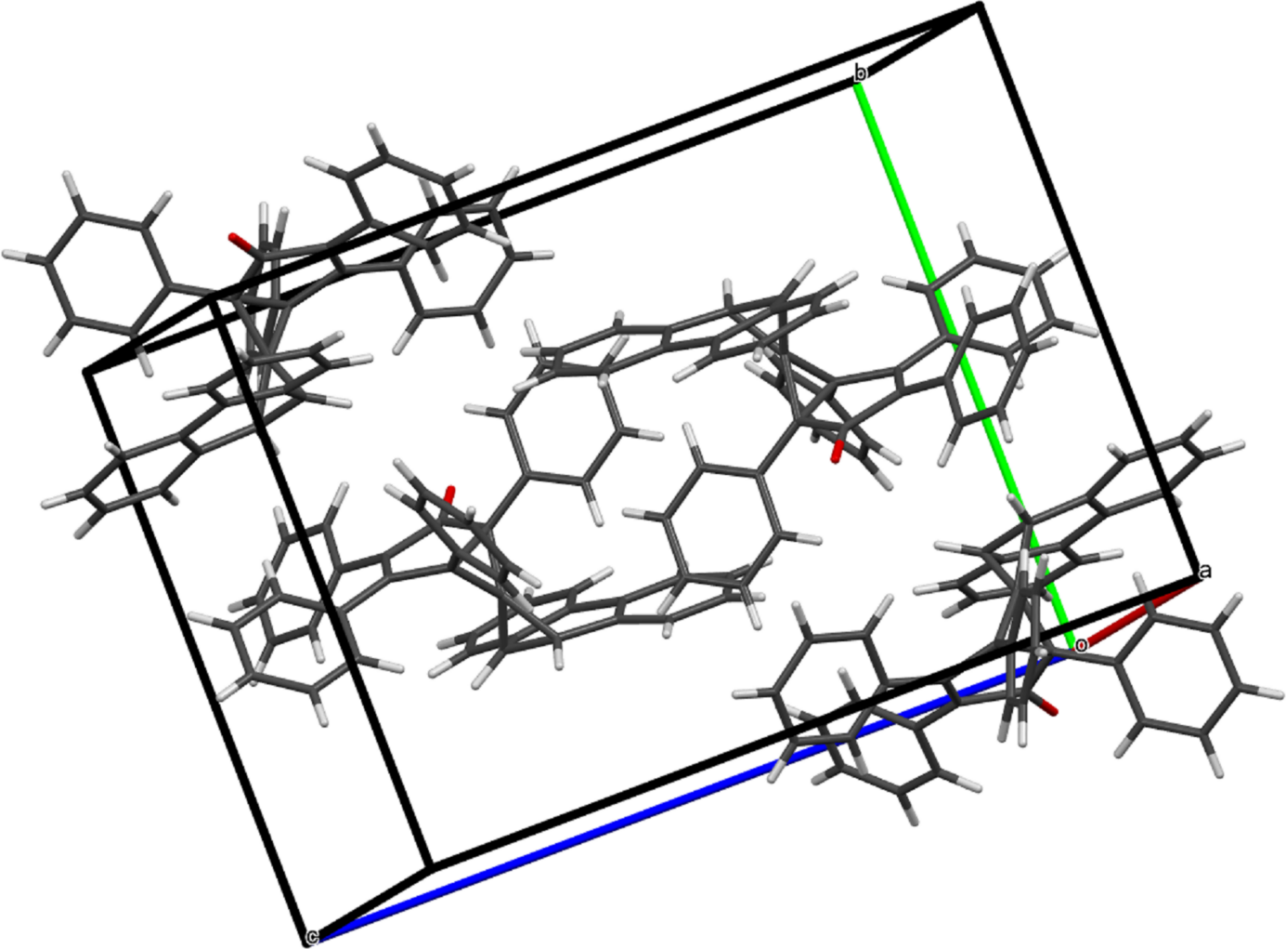
The monoclinic unit cell of **6** contains four mol­ecules.

**Figure 3:**
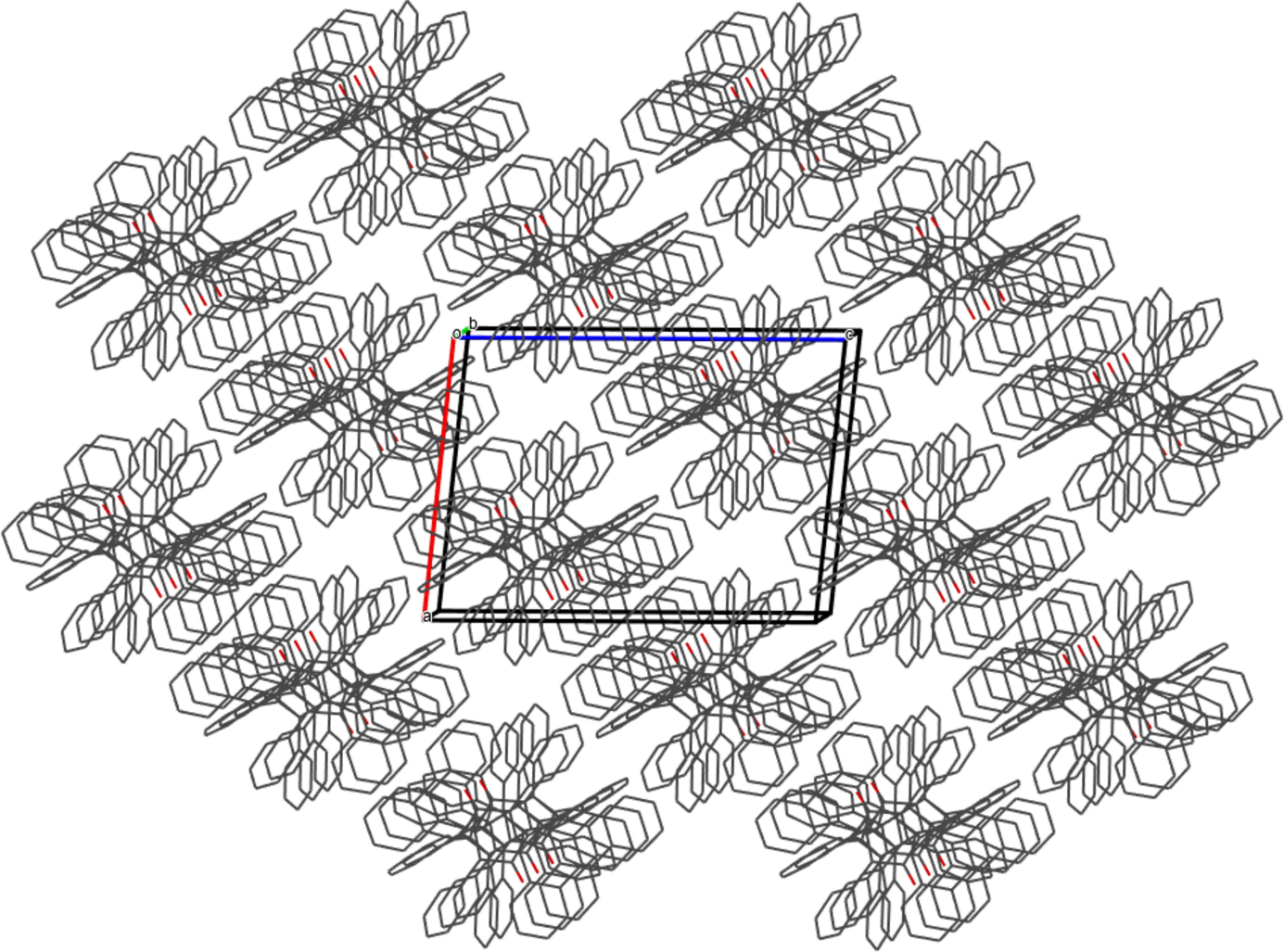
The packing motif of **6** in a 2×2×2 range of cells as viewed with a slight offset along the *b* axis.

The monoclinic unit cell of **6**, with its four mol­ecules, is shown in Fig. 2 ▸. The packing of **6** within a 2×2×2 range of cells, with a slightly offset view along the *b* axis, is displayed in Fig. 3 ▸.

**Figure 4:**
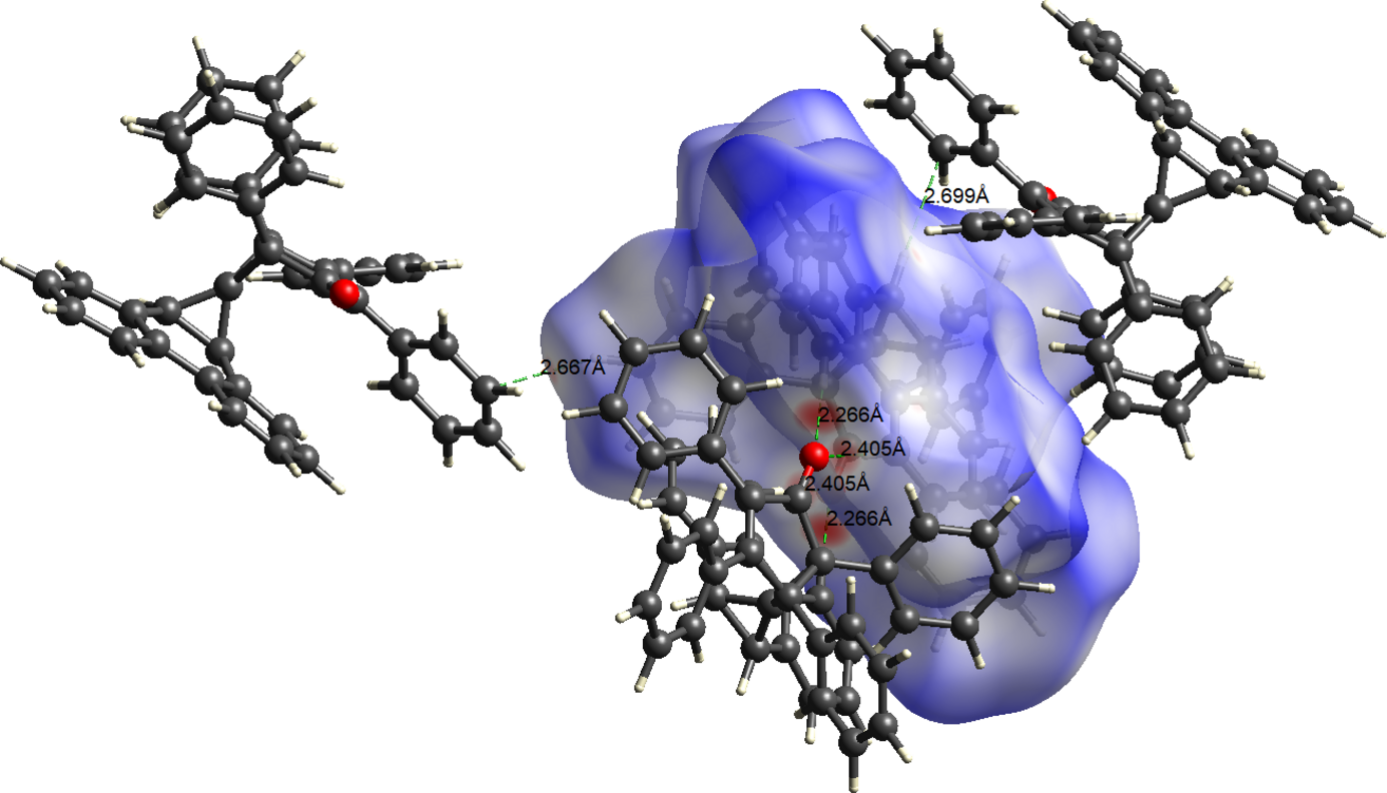
Hirshfield *d*_norm_ surface showing inter­molecular short contacts made by the asymmetric unit in the crystal structure of **6**.

Short inter­molecular contacts within the crystal structure of **6** were also investigated *via* a Hirshfeld surface analysis (Fig. 4 ▸; *CrystalExplorer 21*; Spackman *et al.*, 2021 ▸). The red, grey, and blue regions of the *d*_norm_ surface signify the presence of neighboring atoms at distances less than, approximately equal to, and larger than than the sum of the vdW radii, respectively. Remarkably, as shown in Table 3 ▸, only four such contacts were located (sum of vdW radii − 0.1 Å). Two of these are reciprocal contacts between the carbonyl oxygen and two hydrogen atoms (H6 and H9) in the bay area of the phenanthrene framework of a neighboring mol­ecule to form a dimer. Additional, somewhat weaker, inter­molecular contacts are between C40 and H1, as well as C41 and H24 involving two different and separate neighbors.

**Figure 5:**
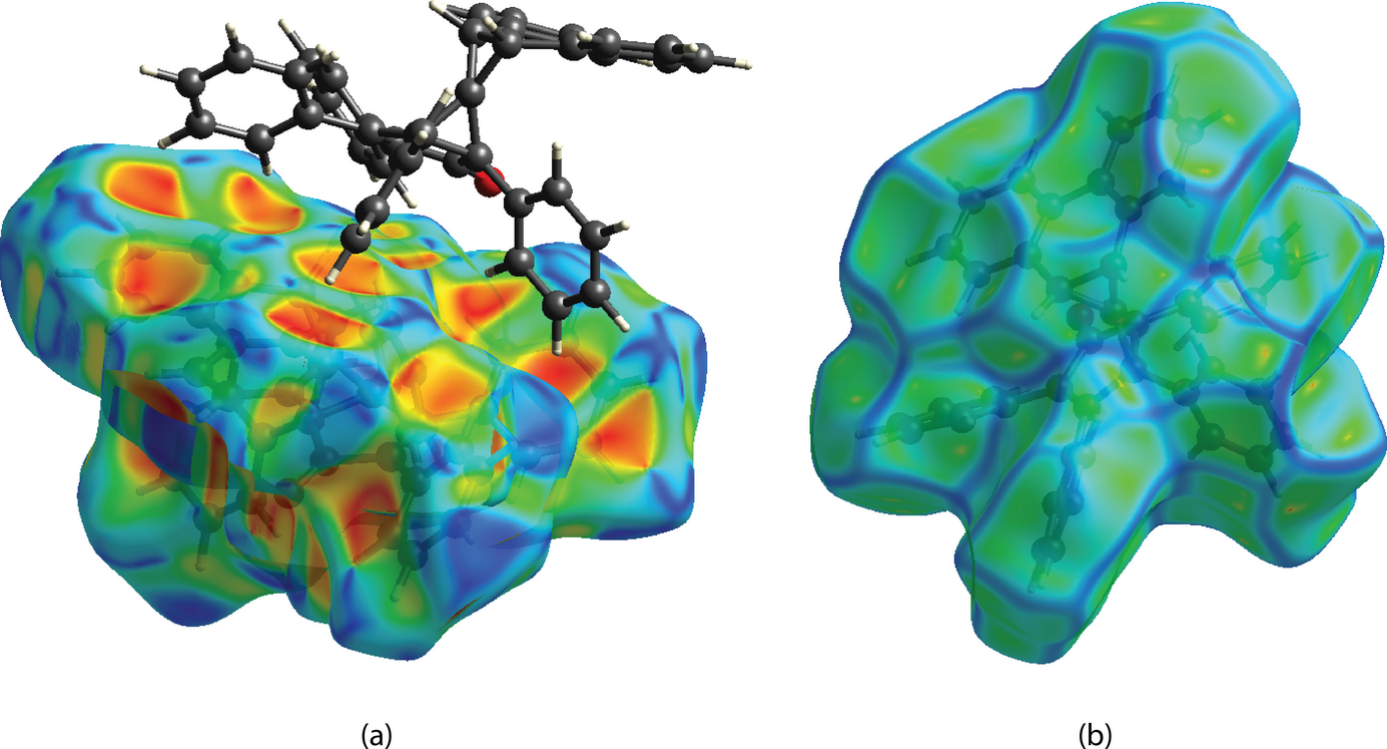
The Hirshfeld surface plotted over (*a*) shape-index and (*b*) curvedness.

The shape-index map of the Hirshfeld surface is shown in Fig. 5 ▸*a*. The map does not show significant red and blue triangles that are conjoined in bow-tie shapes, which are typical of π–π inter­actions. The map does reveal a number of C—H⋯π inter­actions, as evident from the bright-red patches within some of the aryl rings that are complementary to the blue regions of the specific C—H bonds. The curvedness map of the Hirshfeld surface (Fig. 5 ▸*b*) shows numerous smaller planar regions (green) twisted away from one another by ridges (blue). This lack of an extensive planar region on the mol­ecular surface may provide a clue as to why π–π inter­actions are not dominant in the crystal structure of **6**.

**Figure 6:**
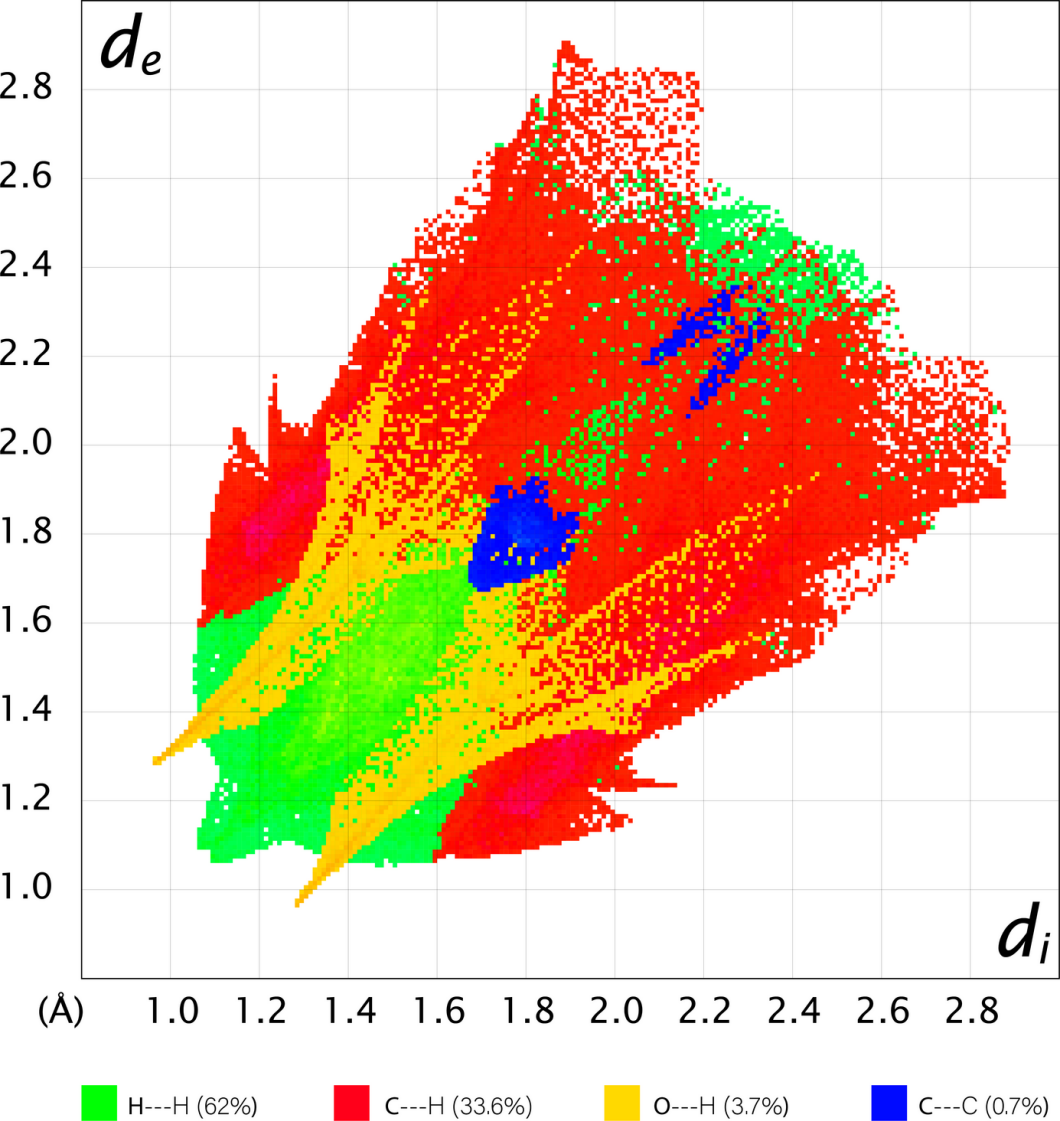
The reciprocal two-dimensional fingerprint plot of *d*_e_*versus d*_i_ for the different types of inter­actions coded by color.

The observations noted above are consistent with the reciprocal 2D fingerprint plot of *d*_e_*vs d*_i_ (where *d*_e_ and *d*_i_ are distances from a given point on the surface to the nearest external and inter­nal atom, respectively), which are shown in Fig. 6 ▸ for specific types of inter­actions such as (*a*) H⋯H, (*b*) C⋯H/H⋯C, (*c*) O⋯H/H⋯O, and (*d*) C⋯C. These maps show that 62% of all inter­actions come from H⋯H which is unsurprising given the large number of hydrogens in the mol­ecule. The C⋯H/H⋯C inter­actions are the second largest contributors (33.6%) followed by O⋯H/H⋯O (3.7%) and C⋯C (0.7%).

## 4. Database survey

A survey of the Cambridge Structural Database (Groom *et al.*, 2016 ▸) using WebCSD (version 1.9.61; accessed April 6, 2025) revealed no previous report of the title compound **6**. The only entry similar to **6** is the phencyclone adduct **3**, which we have recently reported (REFCODE HOJLIF; Roth & Thamattoor, 2024 ▸). To our knowledge these are the only examples in the database in which the central atom of a spiro­pentane moiety is attached to the edges of two separate ring systems.

## 5. Synthesis and crystallization

*Synthesis of exo-1,2,3,5-tetra­phenyl-1a′,9b′-di­hydro­spiro­[bi­cyclo­[3.1.0]hexane-6,1′-cyclo­propa[l]phenanthren]-2-en-4-one* (**6**):

The di­bromo derivative **1** (Nguyen & Thamattoor, 2007 ▸; 0.856 g, 2.45 mmol) was dissolved in THF (30 mL) in a 100 mL three-necked flask under argon atmosphere and stirred with a magnetic stir bar. The solution was cooled to 203 K, and *n*-BuLi (1.2 mL, 2.5 *M* in hexa­nes, 3.0 mmol) was added to the solution. The reaction was allowed to stir in a dry ice/acetone bath for 20 min, and tetra­cyclone (**5**, 0.940 g, 2.44 mmol) in THF (30 mL) was added to the solution slowly over 10 minutes. The solution was kept at 203 K for 2 h, and then allowed to warm to room temperature, where it stirred for the next 14 h. The reaction was quenched with H_2_O (30 mL), the organic layer separated, and the aqueous layer extracted with CH_2_Cl_2_ (3 × 30 mL). The combined organic layers were washed with brine (3 × 30 mL) and dried over anhydrous sodium sulfate. Adduct **6** was isolated as a yellow solid using silica-gel flash-column chromatography (0:100 →15:85 ethyl acetate:hexa­nes). The yield was 189 mg (13%); m.p.: decomposes at 492 K. **6:**^1^H NMR (500 MHz, CDCl_3_) δ: 8.02 (*dd*, *J* = 8.2, 1.3 Hz, 1H), 7.97 (*dd*, *J* = 8.2, 1.1 Hz, 1H), 7.49 (*dd*, *J* = 7.5, 1.4 Hz, 1H), 7.37–7.26 (*m*, 7H), 7.26–7.20 (*m*, 3H), 7.16 (*ddd*, *J* = 8.1, 7.2, 1.4 Hz, 1H), 7.13–7.03 (*m*, 8H), 6.89–6.80 (*m*, 2H), 6.72–6.67 (*m*, 2H), 6.37–6.28 (*m*, 2H), 4.00 (*d*, *J* = 8.5 Hz, 1H), 3.29 (*d*, *J* = 8.5 Hz, 1H). ^13^C NMR (126 MHz, CDCl_3_) δ: 200.1, 166.3, 135.3, 134.9, 134.4, 131.7 (2 carbon resonances), 131.3, 131.1, 131.0, 130.2 (2 carbon resonances), 129.8, 129.4, 129.2, 129.1, 128.8, 128.5, 128.1, 127.9, 127.8, 127.7 (2 carbon resonances), 127.4, 127.1, 127.0, 126.4, 126.1, 123.8, 123.3, 52.1, 49.0, 47.8, 29.5, 24.4. FTIR: ν 3064, 3031, 2987, 2924, 1697, 1597, 1489, 1446 cm^−1^.

## 6. Refinement

Crystal data, data collection and structure refinement details are summarized in Table 4 ▸. H atoms were positioned geometrically (C—H = 0.95 Å) and refined as riding with *U*_iso_(H) = 1.2*U*_eq_(C).

## Supplementary Material

Crystal structure: contains datablock(s) I. DOI: 10.1107/S2056989025004414/yy2017sup1.cif

Structure factors: contains datablock(s) I. DOI: 10.1107/S2056989025004414/yy2017Isup2.hkl

CCDC reference: 2357704

Additional supporting information:  crystallographic information; 3D view; checkCIF report

## Figures and Tables

**Table 1:** Intra­molecular short contacts (Å) in **6** (see Fig. 1 ▸)

Entry number	Site 1	Site 2	Distance
1	O1	Centroid *A*	3.472 (2)
2	O1	C40	2.905 (3)
3	C2	H26	2.562 (2)
4	O1	H40	2.4051 (18)
5	H6	H9	2.05002 (4)

**Table 2:** Normal-to-normal plane angles (°) between the cyclo­pentenone ring and its phenyl substituents in **6** (see Fig. 1 ▸)

Entry number	Color of ring	Angle
1	Green	57.29 (9)
2	Blue	73.67 (10)
3	Magenta	35.06 (9)
4	Orange	39.71 (9)

**Table 3:** Intra­molecular short contacts (Å) in the supra­molecular crystal structure of **6** (see Fig. 4 ▸)

Entry number	Site 1	Site 2	Symmetry operation	Distance
1	O1	H6	2 − *x*, 1 − *y*, 1 − *z*	2.3977 (16)
2	O1	H9	2 − *x*, 1 − *y*, 1 − *z*	2.5362 (18)
3	C40	H1	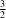 − *x*, − 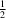 + *y*, 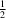 − *z*	2.782 (3)
4	H24	C41	− 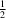 + *x*, 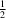 − *y*, 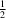 + *z*	2.790 (3)

**Table 4:** Experimental details

Crystal data	
Chemical formula	C_44_H_30_O
*M*_r_	574.68
Crystal system, space group	Monoclinic, *P*2_1_/*n*
Temperature (K)	173
*a*, *b*, *c* (Å)	12.9873 (3), 13.2021 (3), 17.9100 (4)
β (°)	95.796 (1)
*V* (Å^3^)	3055.14 (12)
*Z*	4
Radiation type	Mo *K*α
μ (mm^−1^)	0.07
Crystal size (mm)	0.28 × 0.13 × 0.09

Data collection	
Diffractometer	Bruker D8 Quest Eco
Absorption correction	Multi-scan (*SADABS*; Krause *et al.*, 2015 ▸)
*T*_min_, *T*_max_	0.676, 0.746
No. of measured, independent and observed [*I* > 2σ(*I*)] reflections	67135, 6997, 4365
*R*_int_	0.059
(sin θ/λ)_max_ (Å^−1^)	0.650

Refinement	
*R*[*F*^2^ > 2σ(*F*^2^)], *wR*(*F*^2^), *S*	0.066, 0.208, 1.05
No. of reflections	6997
No. of parameters	406
H-atom treatment	H-atom parameters constrained
Δρ_max_, Δρ_min_ (e Å^−3^)	0.22, −0.26

Computer programs: *APEX4* and *SAINT-Plus* (Bruker, 2021 ▸), *SHELXT* (Sheldrick, 2015*a* ▸), *SHELXL* (Sheldrick, 2015*b* ▸) and *OLEX2* (Dolomanov *et al.*, 2009 ▸).

## supplementary crystallographic information

### exo-1,2,3,5-Tetraphenyl-1a',9b'-dihydrospiro[bicyclo[3.1.0]hexane-6,1'-cyclopropa[l]phenanthren]-2-en-4-one Crystal data

**Table:**

C_44_H_30_O	*F*(000) = 1208
*M**_r_* = 574.68	*D*_x_ = 1.249 Mg m^−^^3^
Monoclinic, *P*2_1_/*n*	Mo *K*α radiation, λ = 0.71073 Å
*a* = 12.9873 (3) Å	Cell parameters from 9968 reflections
*b* = 13.2021 (3) Å	θ = 2.4–27.2°
*c* = 17.9100 (4) Å	µ = 0.07 mm^−^^1^
β = 95.796 (1)°	*T* = 173 K
*V* = 3055.14 (12) Å^3^	Prism, yellow
*Z* = 4	0.28 × 0.13 × 0.09 mm

### exo-1,2,3,5-Tetraphenyl-1a',9b'-dihydrospiro[bicyclo[3.1.0]hexane-6,1'-cyclopropa[l]phenanthren]-2-en-4-one Data collection

**Table:**

Bruker D8 Quest Eco diffractometer	4365 reflections with *I* > 2σ(*I*)
φ and ω scans	*R*_int_ = 0.059
Absorption correction: multi-scan (SADABS; Krause *et al.*, 2015)	θ_max_ = 27.5°, θ_min_ = 2.2°
*T*_min_ = 0.676, *T*_max_ = 0.746	*h* = −16→16
67135 measured reflections	*k* = −17→17
6997 independent reflections	*l* = −23→23

### exo-1,2,3,5-Tetraphenyl-1a',9b'-dihydrospiro[bicyclo[3.1.0]hexane-6,1'-cyclopropa[l]phenanthren]-2-en-4-one Refinement

**Table:**

Refinement on *F*^2^	Primary atom site location: dual
Least-squares matrix: full	Hydrogen site location: inferred from neighbouring sites
*R*[*F*^2^ > 2σ(*F*^2^)] = 0.066	H-atom parameters constrained
*wR*(*F*^2^) = 0.208	*w* = 1/[σ^2^(*F*_o_^2^) + (0.0864*P*)^2^ + 2.5665*P*] where *P* = (*F*_o_^2^ + 2*F*_c_^2^)/3
*S* = 1.05	(Δ/σ)_max_ < 0.001
6997 reflections	Δρ_max_ = 0.22 e Å^−^^3^
406 parameters	Δρ_min_ = −0.26 e Å^−^^3^
0 restraints	

### exo-1,2,3,5-Tetraphenyl-1a',9b'-dihydrospiro[bicyclo[3.1.0]hexane-6,1'-cyclopropa[l]phenanthren]-2-en-4-one Special details

**Table:**

Geometry. All esds (except the esd in the dihedral angle between two l.s. planes) are estimated using the full covariance matrix. The cell esds are taken into account individually in the estimation of esds in distances, angles and torsion angles; correlations between esds in cell parameters are only used when they are defined by crystal symmetry. An approximate (isotropic) treatment of cell esds is used for estimating esds involving l.s. planes.
Refinement. A Bruker D8 Quest Eco diffractometer equipped with a graphite monochromated Mo Kα radiation (λ = 0.71073 Å) and PHOTON 50^TM^ CMOS (complementary metal-oxide semiconductor) detector was used to collect X-ray diffraction data at 173 K with the Bruker Apex 4 suite of programs (Bruker, 2021a). Frames were integrated with a narrow-frame algorithm using the Bruker data reduction software package SAINT+ (Bruker, 2021b) and absorption effects were corrected with the multi-scan method SADABS (Krause *et al.*, 2015). The Olex2 suite of programs (Dolomanov *et al.*, 2009) was used to process data along with the Bruker SHELXTL software package (Sheldrick, 2015a; Sheldrick, 2015b) that was used to perform structure solution by direct methods, and refinement by full-matrix least-squares on F^2^. All nonhydrogen atoms were refined anisotropically with suggested weighting factors and the hydrogens were calculated on a riding model. All cif files were validated with the checkCIF/Platon facility of IUCr that was implemented through Olex 2 (Dolomanov *et al.*, 2009). Hirshfeld surface analysis of the crystal structure was performed with CrystalExplorer 21 (Spackman *et al.*, 2021).

### exo-1,2,3,5-Tetraphenyl-1a',9b'-dihydrospiro[bicyclo[3.1.0]hexane-6,1'-cyclopropa[l]phenanthren]-2-en-4-one Fractional atomic coordinates and isotropic or equivalent isotropic displacement parameters (Å^2^)

**Table:**

	*x*	*y*	*z*	*U*_iso_*/*U*_eq_	
O001	0.90530 (13)	0.39534 (14)	0.34553 (10)	0.0470 (5)	
C002	0.82721 (17)	0.46242 (18)	0.22785 (13)	0.0360 (5)	
C003	0.73177 (17)	0.50140 (17)	0.20571 (13)	0.0349 (5)	
C004	0.67134 (17)	0.52277 (17)	0.27182 (13)	0.0342 (5)	
C005	0.68714 (17)	0.52698 (19)	0.12910 (13)	0.0373 (5)	
C006	0.74121 (17)	0.48192 (18)	0.34187 (13)	0.0347 (5)	
C007	0.93075 (17)	0.64488 (18)	0.43201 (14)	0.0367 (5)	
C008	0.55671 (18)	0.50796 (18)	0.26580 (13)	0.0360 (5)	
C009	0.83527 (17)	0.44040 (18)	0.30974 (13)	0.0361 (5)	
C00A	0.91279 (18)	0.65875 (18)	0.35427 (14)	0.0372 (5)	
C00B	0.72863 (17)	0.59389 (18)	0.32690 (13)	0.0348 (5)	
C00C	0.69744 (17)	0.42527 (18)	0.40306 (13)	0.0363 (5)	
C00D	0.84858 (18)	0.66656 (19)	0.48151 (14)	0.0392 (5)	
C00E	0.91861 (18)	0.45177 (19)	0.18569 (14)	0.0389 (5)	
C00F	0.74928 (18)	0.69608 (18)	0.45090 (14)	0.0397 (6)	
C00G	0.71984 (18)	0.69096 (18)	0.36894 (14)	0.0394 (6)	
H00G	0.662510	0.737146	0.349010	0.047*	
C00H	0.80606 (18)	0.67516 (18)	0.31804 (14)	0.0382 (5)	
H00H	0.799329	0.712636	0.269267	0.046*	
C00I	0.99332 (19)	0.6500 (2)	0.30910 (15)	0.0443 (6)	
H00I	0.980666	0.662551	0.256753	0.053*	
C00J	0.62300 (19)	0.6107 (2)	0.11655 (16)	0.0455 (6)	
H00J	0.607619	0.651789	0.157530	0.055*	
C00K	0.99086 (19)	0.3744 (2)	0.20261 (15)	0.0443 (6)	
H00K	0.978384	0.324605	0.238898	0.053*	
C00L	0.51446 (19)	0.4207 (2)	0.23360 (16)	0.0469 (6)	
H00L	0.558399	0.371015	0.215207	0.056*	
C00M	1.03093 (19)	0.61667 (19)	0.46143 (15)	0.0435 (6)	
H00M	1.044917	0.605358	0.513853	0.052*	
C00N	0.7123 (2)	0.3207 (2)	0.40888 (15)	0.0455 (6)	
H00N	0.750444	0.286924	0.373801	0.055*	
C00O	0.7062 (2)	0.4662 (2)	0.06886 (15)	0.0471 (6)	
H00O	0.749142	0.408185	0.076928	0.057*	
C00P	1.1094 (2)	0.6051 (2)	0.41576 (17)	0.0495 (7)	
H00P	1.176145	0.584513	0.436884	0.059*	
C00Q	0.9405 (2)	0.5246 (2)	0.13299 (15)	0.0471 (6)	
H00Q	0.893362	0.578618	0.121319	0.057*	
C00R	0.8679 (2)	0.6652 (2)	0.56008 (15)	0.0501 (7)	
H00R	0.933771	0.643692	0.582441	0.060*	
C00S	0.6167 (2)	0.3136 (2)	0.51637 (17)	0.0531 (7)	
H00S	0.589290	0.275912	0.554995	0.064*	
C00T	1.0806 (2)	0.3700 (3)	0.16678 (17)	0.0546 (8)	
H00T	1.128716	0.316743	0.178461	0.066*	
C00U	0.6763 (2)	0.7292 (2)	0.49753 (16)	0.0508 (7)	
H00U	0.610890	0.752952	0.475946	0.061*	
C00V	1.0918 (2)	0.6230 (2)	0.33956 (17)	0.0503 (7)	
H00V	1.146582	0.617041	0.308443	0.060*	
C00W	0.6723 (2)	0.2657 (2)	0.46498 (16)	0.0518 (7)	
H00W	0.683118	0.194603	0.468139	0.062*	
C00X	0.3432 (2)	0.4758 (2)	0.25384 (17)	0.0546 (7)	
H00X	0.270578	0.464929	0.249516	0.065*	
C00Y	0.6408 (2)	0.4715 (2)	0.45488 (17)	0.0503 (7)	
H00Y	0.628858	0.542474	0.451848	0.060*	
C00Z	1.0307 (2)	0.5188 (3)	0.09744 (16)	0.0579 (8)	
H00Z	1.044074	0.568314	0.061157	0.069*	
C010	0.49143 (19)	0.5786 (2)	0.29232 (18)	0.0546 (7)	
H010	0.519450	0.638848	0.315118	0.066*	
C011	0.4082 (2)	0.4046 (2)	0.22776 (18)	0.0562 (7)	
H011	0.380162	0.343944	0.205575	0.067*	
C012	0.5814 (2)	0.6344 (3)	0.04445 (18)	0.0600 (8)	
H012	0.538405	0.692357	0.036096	0.072*	
C013	0.6020 (2)	0.5748 (3)	−0.01504 (17)	0.0641 (9)	
H013	0.574255	0.592251	−0.064486	0.077*	
C014	0.6013 (2)	0.4161 (2)	0.51114 (18)	0.0596 (8)	
H014	0.563129	0.449474	0.546430	0.072*	
C015	1.1006 (2)	0.4419 (3)	0.11442 (18)	0.0631 (9)	
H015	1.162277	0.438423	0.090210	0.076*	
C016	0.6629 (2)	0.4895 (3)	−0.00313 (17)	0.0605 (8)	
H016	0.675212	0.446933	−0.044060	0.073*	
C017	0.7935 (2)	0.6942 (3)	0.60555 (17)	0.0597 (8)	
H017	0.808196	0.691085	0.658545	0.072*	
C018	0.6978 (2)	0.7279 (3)	0.57477 (17)	0.0620 (8)	
H018	0.647520	0.749834	0.606197	0.074*	
C019	0.3849 (2)	0.5628 (3)	0.2862 (2)	0.0664 (9)	
H019	0.340823	0.612518	0.304495	0.080*	

### exo-1,2,3,5-Tetraphenyl-1a',9b'-dihydrospiro[bicyclo[3.1.0]hexane-6,1'-cyclopropa[l]phenanthren]-2-en-4-one Atomic displacement parameters (Å^2^)

**Table:**

	*U*^11^	*U*^22^	*U*^33^	*U*^12^	*U*^13^	*U*^23^
O001	0.0373 (9)	0.0563 (11)	0.0461 (10)	0.0146 (8)	−0.0020 (8)	0.0044 (9)
C002	0.0304 (11)	0.0369 (12)	0.0402 (13)	−0.0005 (10)	0.0010 (9)	−0.0003 (10)
C003	0.0314 (11)	0.0352 (12)	0.0373 (12)	−0.0014 (9)	0.0001 (9)	0.0014 (10)
C004	0.0310 (11)	0.0353 (12)	0.0356 (12)	0.0042 (9)	−0.0009 (9)	0.0029 (10)
C005	0.0304 (11)	0.0418 (13)	0.0390 (13)	−0.0037 (10)	0.0002 (9)	0.0032 (11)
C006	0.0286 (11)	0.0377 (12)	0.0370 (12)	0.0014 (9)	0.0000 (9)	0.0027 (10)
C007	0.0319 (12)	0.0330 (12)	0.0441 (13)	−0.0017 (9)	−0.0022 (10)	0.0012 (10)
C008	0.0323 (12)	0.0382 (12)	0.0371 (12)	0.0015 (10)	0.0012 (9)	0.0046 (10)
C009	0.0317 (11)	0.0357 (12)	0.0399 (13)	0.0020 (10)	−0.0015 (10)	−0.0014 (10)
C00A	0.0342 (12)	0.0330 (12)	0.0430 (13)	−0.0026 (10)	−0.0022 (10)	0.0012 (10)
C00B	0.0281 (11)	0.0357 (12)	0.0399 (13)	0.0014 (9)	0.0001 (9)	0.0033 (10)
C00C	0.0295 (11)	0.0384 (12)	0.0402 (13)	0.0009 (10)	0.0001 (9)	0.0044 (10)
C00D	0.0359 (12)	0.0382 (13)	0.0427 (13)	0.0010 (10)	−0.0003 (10)	0.0012 (11)
C00E	0.0326 (12)	0.0439 (13)	0.0399 (13)	−0.0033 (10)	0.0028 (10)	−0.0065 (11)
C00F	0.0365 (12)	0.0375 (12)	0.0442 (13)	0.0018 (10)	0.0004 (10)	−0.0032 (11)
C00G	0.0336 (12)	0.0363 (12)	0.0467 (14)	0.0038 (10)	−0.0040 (10)	−0.0002 (11)
C00H	0.0354 (12)	0.0374 (12)	0.0406 (13)	−0.0023 (10)	−0.0026 (10)	0.0037 (10)
C00I	0.0372 (13)	0.0483 (15)	0.0476 (15)	−0.0062 (11)	0.0051 (11)	−0.0003 (12)
C00J	0.0386 (13)	0.0461 (14)	0.0511 (15)	0.0003 (11)	0.0013 (11)	0.0094 (12)
C00K	0.0366 (13)	0.0476 (14)	0.0475 (14)	−0.0002 (11)	−0.0017 (11)	−0.0115 (12)
C00L	0.0348 (13)	0.0476 (15)	0.0581 (17)	−0.0007 (11)	0.0039 (11)	−0.0070 (13)
C00M	0.0363 (13)	0.0430 (14)	0.0484 (15)	0.0015 (11)	−0.0085 (11)	−0.0030 (11)
C00N	0.0510 (15)	0.0408 (14)	0.0443 (14)	0.0008 (12)	0.0030 (12)	0.0018 (12)
C00O	0.0433 (14)	0.0518 (15)	0.0456 (15)	−0.0032 (12)	0.0010 (11)	−0.0044 (12)
C00P	0.0329 (13)	0.0477 (15)	0.0666 (18)	0.0032 (11)	−0.0019 (12)	−0.0044 (13)
C00Q	0.0429 (14)	0.0548 (16)	0.0435 (14)	−0.0061 (12)	0.0041 (11)	−0.0009 (12)
C00R	0.0454 (15)	0.0606 (17)	0.0427 (14)	0.0011 (13)	−0.0034 (12)	0.0000 (13)
C00S	0.0442 (15)	0.0543 (17)	0.0613 (18)	−0.0015 (13)	0.0073 (13)	0.0221 (14)
C00T	0.0361 (14)	0.0714 (19)	0.0563 (17)	0.0047 (13)	0.0047 (12)	−0.0231 (16)
C00U	0.0416 (14)	0.0568 (17)	0.0541 (16)	0.0062 (13)	0.0045 (12)	−0.0035 (14)
C00V	0.0352 (13)	0.0530 (16)	0.0631 (18)	−0.0025 (12)	0.0072 (12)	−0.0046 (14)
C00W	0.0558 (16)	0.0410 (14)	0.0572 (17)	−0.0044 (13)	−0.0013 (13)	0.0092 (13)
C00X	0.0299 (13)	0.0693 (19)	0.0636 (18)	−0.0053 (13)	0.0003 (12)	−0.0051 (15)
C00Y	0.0439 (14)	0.0451 (15)	0.0647 (18)	0.0092 (12)	0.0189 (13)	0.0135 (13)
C00Z	0.0545 (17)	0.077 (2)	0.0432 (15)	−0.0217 (16)	0.0113 (13)	−0.0096 (15)
C010	0.0306 (13)	0.0502 (16)	0.083 (2)	0.0004 (12)	0.0042 (13)	−0.0159 (15)
C011	0.0453 (15)	0.0571 (17)	0.0655 (19)	−0.0134 (13)	0.0019 (13)	−0.0142 (15)
C012	0.0457 (16)	0.070 (2)	0.0627 (19)	0.0026 (14)	−0.0038 (14)	0.0263 (16)
C013	0.0500 (17)	0.096 (3)	0.0447 (16)	−0.0146 (17)	−0.0056 (13)	0.0216 (17)
C014	0.0509 (16)	0.0635 (19)	0.069 (2)	0.0114 (14)	0.0278 (14)	0.0161 (16)
C015	0.0399 (15)	0.095 (3)	0.0559 (18)	−0.0099 (16)	0.0126 (13)	−0.0251 (18)
C016	0.0504 (16)	0.087 (2)	0.0432 (16)	−0.0170 (16)	0.0014 (13)	−0.0077 (16)
C017	0.0592 (18)	0.077 (2)	0.0427 (15)	0.0018 (16)	0.0020 (13)	−0.0034 (15)
C018	0.0576 (18)	0.077 (2)	0.0524 (17)	0.0048 (16)	0.0122 (14)	−0.0103 (16)
C019	0.0315 (14)	0.067 (2)	0.101 (3)	0.0034 (13)	0.0082 (15)	−0.0238 (19)

### exo-1,2,3,5-Tetraphenyl-1a',9b'-dihydrospiro[bicyclo[3.1.0]hexane-6,1'-cyclopropa[l]phenanthren]-2-en-4-one Geometric parameters (Å, º)

**Table:**

O001—C009	1.214 (3)	C00M—H00M	0.9500
C002—C003	1.364 (3)	C00M—C00P	1.378 (4)
C002—C009	1.489 (3)	C00N—H00N	0.9500
C002—C00E	1.476 (3)	C00N—C00W	1.383 (4)
C003—C004	1.512 (3)	C00O—H00O	0.9500
C003—C005	1.474 (3)	C00O—C016	1.388 (4)
C004—C006	1.568 (3)	C00P—H00P	0.9500
C004—C008	1.495 (3)	C00P—C00V	1.381 (4)
C004—C00B	1.503 (3)	C00Q—H00Q	0.9500
C005—C00J	1.389 (4)	C00Q—C00Z	1.390 (4)
C005—C00O	1.387 (4)	C00R—H00R	0.9500
C006—C009	1.505 (3)	C00R—C017	1.380 (4)
C006—C00B	1.508 (3)	C00S—H00S	0.9500
C006—C00C	1.486 (3)	C00S—C00W	1.380 (4)
C007—C00A	1.400 (3)	C00S—C014	1.369 (4)
C007—C00D	1.482 (3)	C00T—H00T	0.9500
C007—C00M	1.404 (3)	C00T—C015	1.378 (5)
C008—C00L	1.377 (4)	C00U—H00U	0.9500
C008—C010	1.376 (4)	C00U—C018	1.384 (4)
C00A—C00H	1.486 (3)	C00V—H00V	0.9500
C00A—C00I	1.390 (3)	C00W—H00W	0.9500
C00B—C00G	1.496 (3)	C00X—H00X	0.9500
C00B—C00H	1.490 (3)	C00X—C011	1.376 (4)
C00C—C00N	1.396 (3)	C00X—C019	1.373 (4)
C00C—C00Y	1.383 (4)	C00Y—H00Y	0.9500
C00D—C00F	1.405 (3)	C00Y—C014	1.385 (4)
C00D—C00R	1.404 (4)	C00Z—H00Z	0.9500
C00E—C00K	1.399 (4)	C00Z—C015	1.375 (5)
C00E—C00Q	1.396 (4)	C010—H010	0.9500
C00F—C00G	1.481 (3)	C010—C019	1.393 (4)
C00F—C00U	1.396 (4)	C011—H011	0.9500
C00G—H00G	1.0000	C012—H012	0.9500
C00G—C00H	1.528 (3)	C012—C013	1.373 (5)
C00H—H00H	1.0000	C013—H013	0.9500
C00I—H00I	0.9500	C013—C016	1.380 (5)
C00I—C00V	1.385 (4)	C014—H014	0.9500
C00J—H00J	0.9500	C015—H015	0.9500
C00J—C012	1.384 (4)	C016—H016	0.9500
C00K—H00K	0.9500	C017—H017	0.9500
C00K—C00T	1.387 (4)	C017—C018	1.380 (4)
C00L—H00L	0.9500	C018—H018	0.9500
C00L—C011	1.390 (4)	C019—H019	0.9500
			
C003—C002—C009	109.4 (2)	C011—C00L—H00L	119.7
C003—C002—C00E	129.9 (2)	C007—C00M—H00M	119.3
C00E—C002—C009	120.3 (2)	C00P—C00M—C007	121.3 (2)
C002—C003—C004	111.8 (2)	C00P—C00M—H00M	119.3
C002—C003—C005	128.2 (2)	C00C—C00N—H00N	119.5
C005—C003—C004	120.0 (2)	C00W—C00N—C00C	121.0 (3)
C003—C004—C006	104.97 (18)	C00W—C00N—H00N	119.5
C008—C004—C003	120.7 (2)	C005—C00O—H00O	119.8
C008—C004—C006	120.30 (19)	C005—C00O—C016	120.5 (3)
C008—C004—C00B	123.5 (2)	C016—C00O—H00O	119.8
C00B—C004—C003	111.65 (19)	C00M—C00P—H00P	119.7
C00B—C004—C006	58.78 (15)	C00M—C00P—C00V	120.6 (2)
C00J—C005—C003	120.4 (2)	C00V—C00P—H00P	119.7
C00O—C005—C003	120.5 (2)	C00E—C00Q—H00Q	119.6
C00O—C005—C00J	119.1 (2)	C00Z—C00Q—C00E	120.7 (3)
C009—C006—C004	104.23 (18)	C00Z—C00Q—H00Q	119.6
C009—C006—C00B	111.50 (19)	C00D—C00R—H00R	119.3
C00B—C006—C004	58.47 (14)	C017—C00R—C00D	121.5 (3)
C00C—C006—C004	121.97 (19)	C017—C00R—H00R	119.3
C00C—C006—C009	119.0 (2)	C00W—C00S—H00S	120.3
C00C—C006—C00B	125.6 (2)	C014—C00S—H00S	120.3
C00A—C007—C00D	120.8 (2)	C014—C00S—C00W	119.3 (3)
C00A—C007—C00M	117.6 (2)	C00K—C00T—H00T	119.6
C00M—C007—C00D	121.5 (2)	C015—C00T—C00K	120.7 (3)
C00L—C008—C004	119.4 (2)	C015—C00T—H00T	119.6
C010—C008—C004	122.0 (2)	C00F—C00U—H00U	119.6
C010—C008—C00L	118.5 (2)	C018—C00U—C00F	120.9 (3)
O001—C009—C002	126.2 (2)	C018—C00U—H00U	119.6
O001—C009—C006	124.8 (2)	C00I—C00V—H00V	120.4
C002—C009—C006	109.06 (19)	C00P—C00V—C00I	119.1 (3)
C007—C00A—C00H	120.5 (2)	C00P—C00V—H00V	120.4
C00I—C00A—C007	120.5 (2)	C00N—C00W—H00W	119.9
C00I—C00A—C00H	118.8 (2)	C00S—C00W—C00N	120.3 (3)
C004—C00B—C006	62.75 (15)	C00S—C00W—H00W	119.9
C00G—C00B—C004	143.8 (2)	C011—C00X—H00X	120.4
C00G—C00B—C006	139.7 (2)	C019—C00X—H00X	120.4
C00H—C00B—C004	132.4 (2)	C019—C00X—C011	119.1 (2)
C00H—C00B—C006	131.5 (2)	C00C—C00Y—H00Y	119.5
C00H—C00B—C00G	61.54 (16)	C00C—C00Y—C014	121.1 (3)
C00N—C00C—C006	119.5 (2)	C014—C00Y—H00Y	119.5
C00Y—C00C—C006	122.8 (2)	C00Q—C00Z—H00Z	119.8
C00Y—C00C—C00N	117.7 (2)	C015—C00Z—C00Q	120.4 (3)
C00F—C00D—C007	120.5 (2)	C015—C00Z—H00Z	119.8
C00R—C00D—C007	122.0 (2)	C008—C010—H010	119.5
C00R—C00D—C00F	117.3 (2)	C008—C010—C019	120.9 (3)
C00K—C00E—C002	121.0 (2)	C019—C010—H010	119.5
C00Q—C00E—C002	120.6 (2)	C00L—C011—H011	119.7
C00Q—C00E—C00K	118.1 (2)	C00X—C011—C00L	120.5 (3)
C00D—C00F—C00G	120.6 (2)	C00X—C011—H011	119.7
C00U—C00F—C00D	120.4 (2)	C00J—C012—H012	119.8
C00U—C00F—C00G	119.0 (2)	C013—C012—C00J	120.4 (3)
C00B—C00G—H00G	115.7	C013—C012—H012	119.8
C00B—C00G—C00H	59.01 (16)	C012—C013—H013	120.0
C00F—C00G—C00B	120.8 (2)	C012—C013—C016	120.1 (3)
C00F—C00G—H00G	115.7	C016—C013—H013	120.0
C00F—C00G—C00H	117.8 (2)	C00S—C014—C00Y	120.6 (3)
C00H—C00G—H00G	115.7	C00S—C014—H014	119.7
C00A—C00H—C00B	117.3 (2)	C00Y—C014—H014	119.7
C00A—C00H—C00G	117.8 (2)	C00T—C015—H015	120.2
C00A—C00H—H00H	116.7	C00Z—C015—C00T	119.6 (3)
C00B—C00H—C00G	59.44 (16)	C00Z—C015—H015	120.2
C00B—C00H—H00H	116.7	C00O—C016—H016	120.1
C00G—C00H—H00H	116.7	C013—C016—C00O	119.8 (3)
C00A—C00I—H00I	119.6	C013—C016—H016	120.1
C00V—C00I—C00A	120.8 (3)	C00R—C017—H017	119.7
C00V—C00I—H00I	119.6	C00R—C017—C018	120.6 (3)
C005—C00J—H00J	119.9	C018—C017—H017	119.7
C012—C00J—C005	120.2 (3)	C00U—C018—H018	120.4
C012—C00J—H00J	119.9	C017—C018—C00U	119.1 (3)
C00E—C00K—H00K	119.8	C017—C018—H018	120.4
C00T—C00K—C00E	120.5 (3)	C00X—C019—C010	120.2 (3)
C00T—C00K—H00K	119.8	C00X—C019—H019	119.9
C008—C00L—H00L	119.7	C010—C019—H019	119.9
C008—C00L—C011	120.7 (3)		
			
C002—C003—C004—C006	−4.7 (3)	C009—C006—C00C—C00N	−26.6 (3)
C002—C003—C004—C008	−144.7 (2)	C009—C006—C00C—C00Y	154.2 (2)
C002—C003—C004—C00B	57.2 (3)	C00A—C007—C00D—C00F	3.5 (4)
C002—C003—C005—C00J	−143.8 (3)	C00A—C007—C00D—C00R	−172.2 (2)
C002—C003—C005—C00O	38.0 (4)	C00A—C007—C00M—C00P	1.3 (4)
C002—C00E—C00K—C00T	−174.8 (2)	C00A—C00I—C00V—C00P	−0.1 (4)
C002—C00E—C00Q—C00Z	175.0 (2)	C00B—C004—C006—C009	−106.7 (2)
C003—C002—C009—O001	171.5 (2)	C00B—C004—C006—C00C	114.9 (3)
C003—C002—C009—C006	−7.7 (3)	C00B—C004—C008—C00L	−158.5 (2)
C003—C002—C00E—C00K	−151.5 (3)	C00B—C004—C008—C010	20.7 (4)
C003—C002—C00E—C00Q	34.9 (4)	C00B—C006—C009—O001	124.0 (3)
C003—C004—C006—C009	−0.2 (2)	C00B—C006—C009—C002	−56.8 (2)
C003—C004—C006—C00B	106.5 (2)	C00B—C006—C00C—C00N	177.9 (2)
C003—C004—C006—C00C	−138.6 (2)	C00B—C006—C00C—C00Y	−1.4 (4)
C003—C004—C008—C00L	46.1 (3)	C00B—C00G—C00H—C00A	−106.9 (2)
C003—C004—C008—C010	−134.7 (3)	C00C—C006—C009—O001	−34.8 (3)
C003—C004—C00B—C006	−94.8 (2)	C00C—C006—C009—C002	144.4 (2)
C003—C004—C00B—C00G	126.7 (3)	C00C—C006—C00B—C004	−109.0 (2)
C003—C004—C00B—C00H	27.8 (3)	C00C—C006—C00B—C00G	33.8 (4)
C003—C005—C00J—C012	179.9 (2)	C00C—C006—C00B—C00H	127.2 (3)
C003—C005—C00O—C016	179.1 (2)	C00C—C00N—C00W—C00S	0.0 (4)
C004—C003—C005—C00J	33.2 (3)	C00C—C00Y—C014—C00S	−0.6 (5)
C004—C003—C005—C00O	−145.0 (2)	C00D—C007—C00A—C00H	−12.4 (3)
C004—C006—C009—O001	−174.7 (2)	C00D—C007—C00A—C00I	172.3 (2)
C004—C006—C009—C002	4.5 (2)	C00D—C007—C00M—C00P	−174.4 (2)
C004—C006—C00B—C00G	142.8 (3)	C00D—C00F—C00G—C00B	55.7 (3)
C004—C006—C00B—C00H	−123.9 (3)	C00D—C00F—C00G—C00H	−13.0 (3)
C004—C006—C00C—C00N	106.0 (3)	C00D—C00F—C00U—C018	−3.9 (4)
C004—C006—C00C—C00Y	−73.2 (3)	C00D—C00R—C017—C018	−1.4 (5)
C004—C008—C00L—C011	179.5 (3)	C00E—C002—C003—C004	−165.0 (2)
C004—C008—C010—C019	−179.8 (3)	C00E—C002—C003—C005	12.2 (4)
C004—C00B—C00G—C00F	130.1 (3)	C00E—C002—C009—O001	−15.0 (4)
C004—C00B—C00G—C00H	−124.0 (4)	C00E—C002—C009—C006	165.8 (2)
C004—C00B—C00H—C00A	−113.8 (3)	C00E—C00K—C00T—C015	0.5 (4)
C004—C00B—C00H—C00G	138.5 (3)	C00E—C00Q—C00Z—C015	−0.9 (4)
C005—C003—C004—C006	177.9 (2)	C00F—C00D—C00R—C017	−1.7 (4)
C005—C003—C004—C008	37.8 (3)	C00F—C00G—C00H—C00A	4.2 (3)
C005—C003—C004—C00B	−120.2 (2)	C00F—C00G—C00H—C00B	111.1 (2)
C005—C00J—C012—C013	0.9 (4)	C00F—C00U—C018—C017	0.7 (5)
C005—C00O—C016—C013	1.1 (4)	C00G—C00B—C00H—C00A	107.8 (2)
C006—C004—C008—C00L	−88.0 (3)	C00G—C00F—C00U—C018	175.1 (3)
C006—C004—C008—C010	91.3 (3)	C00H—C00A—C00I—C00V	−172.5 (2)
C006—C004—C00B—C00G	−138.4 (4)	C00H—C00B—C00G—C00F	−106.0 (2)
C006—C004—C00B—C00H	122.6 (3)	C00I—C00A—C00H—C00B	115.6 (3)
C006—C00B—C00G—C00F	15.8 (4)	C00I—C00A—C00H—C00G	−176.4 (2)
C006—C00B—C00G—C00H	121.7 (3)	C00J—C005—C00O—C016	0.9 (4)
C006—C00B—C00H—C00A	−24.9 (4)	C00J—C012—C013—C016	1.2 (5)
C006—C00B—C00H—C00G	−132.7 (3)	C00K—C00E—C00Q—C00Z	1.3 (4)
C006—C00C—C00N—C00W	−179.8 (2)	C00K—C00T—C015—C00Z	−0.1 (4)
C006—C00C—C00Y—C014	180.0 (3)	C00L—C008—C010—C019	−0.6 (5)
C007—C00A—C00H—C00B	−59.7 (3)	C00M—C007—C00A—C00H	171.9 (2)
C007—C00A—C00H—C00G	8.3 (3)	C00M—C007—C00A—C00I	−3.4 (4)
C007—C00A—C00I—C00V	2.8 (4)	C00M—C007—C00D—C00F	179.1 (2)
C007—C00D—C00F—C00G	9.5 (4)	C00M—C007—C00D—C00R	3.4 (4)
C007—C00D—C00F—C00U	−171.6 (2)	C00M—C00P—C00V—C00I	−2.0 (4)
C007—C00D—C00R—C017	174.1 (3)	C00N—C00C—C00Y—C014	0.7 (4)
C007—C00M—C00P—C00V	1.3 (4)	C00O—C005—C00J—C012	−1.9 (4)
C008—C004—C006—C009	140.1 (2)	C00Q—C00E—C00K—C00T	−1.1 (4)
C008—C004—C006—C00B	−113.2 (2)	C00Q—C00Z—C015—C00T	0.3 (4)
C008—C004—C006—C00C	1.7 (3)	C00R—C00D—C00F—C00G	−174.6 (2)
C008—C004—C00B—C006	107.8 (2)	C00R—C00D—C00F—C00U	4.4 (4)
C008—C004—C00B—C00G	−30.6 (5)	C00R—C017—C018—C00U	2.0 (5)
C008—C004—C00B—C00H	−129.6 (3)	C00U—C00F—C00G—C00B	−123.3 (3)
C008—C00L—C011—C00X	0.2 (5)	C00U—C00F—C00G—C00H	168.0 (2)
C008—C010—C019—C00X	0.4 (5)	C00W—C00S—C014—C00Y	0.1 (5)
C009—C002—C003—C004	7.7 (3)	C00Y—C00C—C00N—C00W	−0.5 (4)
C009—C002—C003—C005	−175.1 (2)	C010—C008—C00L—C011	0.3 (4)
C009—C002—C00E—C00K	36.5 (3)	C011—C00X—C019—C010	0.1 (5)
C009—C002—C00E—C00Q	−137.1 (2)	C012—C013—C016—C00O	−2.2 (5)
C009—C006—C00B—C004	93.9 (2)	C014—C00S—C00W—C00N	0.2 (4)
C009—C006—C00B—C00G	−123.3 (3)	C019—C00X—C011—C00L	−0.4 (5)
C009—C006—C00B—C00H	−30.0 (3)		
